# Supplementary material for: SyNDI: synchronous network data integration framework
Source: BMC Bioinformatics. 2018 Nov 6;19:403. doi: 10.1186/s12859-018-2426-5 (PMC6219086; doi:10.1186/s12859-018-2426-5)
Supplement: Supplementary file 5 — Files of the example presented in Additional file 4. This additional file is a zip package that contains all files used in Additional file 4. Detailed descriptions of each of these files are presented in a README file included in this zip package. (GZ 30411 kb) [file 12859_2018_2426_MOESM5_ESM.gz › SyNDITools/Meme2Fimo/meme_4.9.0_4.tar.gz!/meme_4.9.0/website/cgi-bin/db_list_view.tmpl]

<!-- TMPL\_VAR NAME=title --> - Documentation 


# MEME Suite

The MEME Suite web application requires the use of JavaScript  
Javascript doesn't seem to be available on your browser.


---

# available for search

---

### The databases that can be searched are grouped into categories:

- ### ">


---

">


---


(protein and nucleotide)(nucleotide only)(protein only)


##### Comparative database(s):


---
